# Knowledge and attitude toward human papillomavirus vaccination and associated factors among adolescent school girls in Ambo town, Ethiopia, 2021: A multicenter cross‐sectional study

**DOI:** 10.1002/hsr2.1305

**Published:** 2023-05-31

**Authors:** Ephrem Yohannes, Mulugeta W. Beyen, Gizachew A. Bulto, Eshetu E. Chaka, Bikila T. Debelo, Maru M. Erena, Tefera L. Tasu

**Affiliations:** ^1^ Department of Midwifery Ambo University Ambo Ethiopia; ^2^ Reproductive Health division South West Shewa Zone Health Office Waliso Ethiopia; ^3^ Department of Public Health, Epidimiology and Biostatistics Unit, College of Medicine and Health Sciences Ambo University Ambo Ethiopia; ^4^ Department of Nursing, College of Medicine and Health Sciences Ambo University Ambo Ethiopia

**Keywords:** adolescent girls, attitude, human papillomavirus vaccine, knowledge

## Abstract

**Background and Aims:**

The human papillomavirus (HPV) vaccination has shown considerable promise in lowering the incidence of cervical cancer. However, there are few studies done on knowledge and attitude toward the HPV vaccine, especially in the local setting. Therefore, this study aimed to assess knowledge and attitudes regarding HPV vaccination and related factors among adolescent girls in Ambo town, Ethiopia, in 2021.

**Methods:**

A cross‐sectional study was conducted from May 1 to May 30, 2021, at selected schools in Ambo town among 422 adolescent girls. A structured self‐administered questionnaire was used to collect the data. The collected data were coded, and entered into Epi‐Info 7.2.2.6 and exported to STATA 16 for analysis. Descriptive statistics were used to compute summary statistics and proportions. Bivariate and multivariate logistic regression analyses were carried out. The adjusted odds ratio (AOR) along with a 95% confidence interval (CI) was computed to ascertain the association.

**Results:**

Current study revealed that 24.9% (95% CI: 20.8–29.0) and 55.6% (95% CI: 50.5–60.4) of respondents had good knowledge and favorable attitude, respectively. The factors like being secondary school students [AOR = 2.01, 95% CI; (1.24, 3.27)], having health workers as a source of information [AOR = 2.13, 95% CI; (1.30, 3.47)], and respondents who have heard about the HPV vaccine at school [AOR = 1.66, 95% CI; (1.02, 2.71)] were significantly associated with respondent's knowledge of HPV vaccine. Moreover, the perceived severity of the diseases [AOR = 2.89, 95% CI; (1.61, 5.21)], and the perceived benefit of the vaccine [AOR = 4.26, 95% CI; (2.43, 7.48)] were factors significantly associated with an attitude of the respondents about the HPV vaccine.

**Conclusion:**

There was low knowledge and unfavorable attitude toward HPV vaccination among the school adolescents in the study area. Several seemingly major deterrents of knowledge and attitude toward the HPV vaccination were identified. Therefore, all parties involved should collaborate to improve female students' knowledge and attitudes toward Human Papilloma Virus vaccination.

## 1 BACKGROUND

The human papillomaviruses (HPVs) are a large family of tiny, nonenveloped, double‐stranded, circular RNA viruses that are extremely prevalent. As sexual contact begins, both men and women are quickly exposed to it.^1^, ^2^ The nononcogenic HPV types 6 and 11 are thought to be the main sources of 90% of genital warts, whereas strains 16 and 18 of HPV are the most common causes of cervical cancer (CC).^3^ Each year, there are around 560,000 new cases of CC and about 275,000 deaths, with more than 80% taking place in underdeveloped nations.^3^, ^4^

A woman dies from CC every 2 min. This is the worst and a fourth leading cause of death, continues to affect approximately 570,000 women with around 311,000 deaths worldwide. In East Africa, it is estimated that CC affects 42.7 cases per 100,000 women and causes the death of 35 per 100,000 women.^5^ CC is the second most common cancer among women in Ethiopia with an estimated annual death toll of 4884 and 7095 women are diagnosed with it.^6^ More than 85% of those affected by HPV are young, uneducated women living in the world's poorest countries. Moreover, many of them were also mothers of young children whose survival was later cut short by the untimely death of their mothers.^5^

The HPV vaccine is a prophylactic vaccine developed to prevent the most common high‐risk strains and low‐risk infections of HPV.^7^ The bivalent HPV vaccine was introduced in Ethiopia on December 3, 2018 for 14‐year‐old girls.^8^ To reach all 14‐year‐old females, the vaccine is presently predominantly administered through a school‐based strategy.^9^ However, obstacles to a successful HPV vaccination program in Ethiopia included social influence, a lack of understanding about CC and screening, and a lack of health education regarding the HPV vaccine.^10^

A lack of understanding about the HPV vaccine was one cause of morbidity and mortality associated with the HPV. According to certain research, the majority of adolescents are unaware of the HPV vaccine, which is a significant obstacle to vaccination.^11^, ^12^ Most teens have a misunderstanding about the HPV vaccine, making it difficult to accept the vaccination.^12^, ^13^ Moreover, the successful implementation of a vaccination program is hampered by various key demographic factors like, age, educational level, occupation, and residence.^14^

On the other hand, the acceptability of the HPV vaccine was affected largely due to unfavorable attitudes and beliefs toward the vaccine.^15^, ^16^ It was also shown that the reason for not being vaccinated was a delay, as well as a refusal based on the belief that vaccines were unnecessary.^17^ These negative attitudes (hesitations) are not limited to those who refused vaccination but also to those who encourage others to refuse vaccination. As a result, many healthcare professionals have reported increasing difficulties to develop trusting relationships with parents and adolescents.^18^, ^19^

Poor knowledge and unfavorable attitudes have an impact not only on adolescents but also on others. Due to a lack of understanding and negative attitudes, 87.3% of teenagers did not have their parents or family support to be immunized.^15^ According to data from the Centers for Disease Control and Prevention (CDC), most parents are unwilling to vaccinate their children due to a lack of awareness about the vaccine.^20^ Most parents often delay the decision to get the HPV vaccine because they think their child was at low risk for HPV infection.^15^, ^20^ Thus, the respondent's negative perceptions, unfavorable attitudes as well as poor knowledge about the HPV vaccine impacted the implementation of the vaccination negatively.^21^

The HPV vaccine has only recently been introduced in Ethiopia, and there have been relatively few studies looking at knowledge, attitude toward the HPV vaccine, and associated factors among adolescents. Understanding the level of knowledge and attitude of adolescents about the HPV vaccine and related issues may be crucial for the vaccination program to be effective and the implementation of a good and well‐respected primary preventive program for CC. Thus, this study aimed to assess HPV vaccine knowledge, attitude, and the associated factors among adolescent girls in Ambo town, Ethiopia.

## 2 METHODS

### 2.1 Study setting, period, and design

The study was conducted in the town of Ambo, West Shewa zone, Oromia regional state, Ethiopia from May 1 to May 30, 2021. The town of Ambo is located approximately 114 km away to the West of Addis Ababa, the capital of Ethiopia. The city has six administrative units (small administrative units) with an estimated total population of 2,381,079, of which 1,214,350 are women. It has 16 primary schools, including 9 public schools and 7 private schools, and a total of 6 secondary schools, including 5 public schools and 1 private school. The number of adolescent girls enrolled in all public and private schools were 11,250 in 2020/2021. Based on the report from the Ambo town health office, 2021, the first round of HPV vaccination began at all primary schools for 14‐year‐old girls in 2018. This cross‐sectional study design was carried out to conduct the study among adolescent girls aged 14–19 years old from randomly selected schools in Ambo town.

### 2.2 Sample size determination and sampling procedure

#### 2.2.1 Sample size determination

Because no studies in this area had been found at the time of the study, the sample size was computed using the single population proportion formula, based on the assumption of a proportion (P) of 50% that yields a maximum sample size. Moreover, Zα/2 = 1.96, the margin of error (*d*) = 5%, and the α value 0.05 were used. Therefore, assuming 10% as the nonresponse rate, the final total sample size was 422 study participants. Eight primary schools and 3 high schools were chosen at random from among the 16 primary schools and 6 high schools in the town of Ambo. The sample size was distributed proportionally to each selected school. Finally, study participants were selected from each school by using a simple random sampling technique with a computer‐generated random number method.

#### 2.2.2 Operational definitions

##### Adolescence

The age between 10 and 19 years was considered an adolescent girl.^22^

##### Knowledge

There were three subsections of knowledge‐related questions, specifically related to CC, HPV infection, and HPV vaccine with a maximum possible score of 32. The knowledge of CC, knowledge of HPV Infection, and the knowledge of HPV vaccine have 12, 13, and 7 knowledge‐related questions, respectively. The total score was calculated and those with mean and below mean scores were considered as having poor overall knowledge, while those with the above mean score were displayed as having good overall knowledge. The same approach was used for knowledge of CC, knowledge of HPV infection, and overall knowledge.^19^

##### Attitude

A total of seven items with a 5‐point Likert scale was developed to measure attitudes toward the HPV vaccine. It was awarded 5 points for “totally agree” and reduced to 1 point for “totally disagree.” Then, attitude scores were calculated for each participant, ranging from 0 to 7. Finally, a composite attitude variable was generated from the score using the mean as the cut‐off point when checking for the normality of the score. Therefore, participants with above‐mean scores were classified as having “favorable attitudes,” while those with mean and below‐mean scores were classified as having “unfavorable attitudes.”^23^

##### Perception

A total of 13 items were constructed for all sections of perception. Perception‐related questions included the susceptibility of the diseases, the severity of the diseases, benefits of the HPV vaccine, and the barriers to HPV vaccination. As a result, each category has three items unless barriers to HPV vaccination which has four items. The available responses were noted on a Likert scale. Therefore, a score below the mean indicates a stronger belief in refusing vaccination and was labeled as “Poor Perception” while a score above the average was considered to be “Good Perception.”^23^

#### 2.2.3 Independent variables

Independent factors were sociodemographic parameters (age, educational status, occupation, and place of residence), perception (perceived susceptibility, perceived severity, perceived benefit, and perceived barriers), and HPV vaccine promotion.

#### 2.2.4 Dependent variables

Knowledge and attitude toward HPV vaccine.

#### 2.2.5 Data collection tool and procedure

The questionnaire was prepared in the English language that was translated into Afaan Oromo and then translated back into English to check its consistency. The data collection tool has been adapted through a review of different pieces of literatures^3^ and similar previous studies.^9^, ^11^, ^16^, ^24^ An eight‐part questionnaire was developed to assess sociodemographic characteristics, knowledge about CC, knowledge about HPV infection and vaccination, attitudes toward HPV vaccination, the presence of HPV vaccination advertisements, and source of information. A self‐administered Afaan Oromo version questionnaire was used to collect the data. The data were collected by eight experienced bachelor's degrees in nursing. On top of that, eight teachers were selected to facilitate the data collection process and three master's degree holder senior midwives were supervised the data collection process.

### 2.3 Data quality assurance

Data collectors and supervisors received 2 days of training on the objectives of the study, the relevance of the study, confidentiality, respondent's rights, informed consent, and interview techniques before the actual data collection period. The questionnaire was pretested on 5% of the samples at Ginchi high school to ensure the accuracy of the responses, the clarity of the language, and the applicability of the tool. Cronbach's α was used to assess the tool's internal consistency and validity with the value of 0.84. This proved that the survey was credible and legitimate.^25^ The principal investigator and supervisors carried out on‐site checks and reviews of all completed questionnaires to ensure the completeness and consistency of collected data. The principal investigator double‐checked the entered data regularly to ensure that it was accurate.

### 2.4 Data processing and analysis

After data collection, each questionnaire was checked for completeness and entered into Ep‐info version 7.2.2.6. Then, the data were exported to STATA version 16 for cleaning, coding, and final analysis. The data were checked for missed values and outliers. Descriptive analysis (like frequencies, tables, percentages, means, and standard deviation) was done to describe the required variable. There were no outliers and missing data. We confirmed that the assumptions of the analysis were met. The variables with a *p* value of less than 0.25 on bivariate logistic regression with crude odds ratio (COR) at 95% confidence interval (CI) were considered to be the candidates for the multivariable logistic regression model. This study used the variable selection process by the backward‐stepwise model during the initial stage of developing the model. Multivariable logistic regression with adjusted odds ratio (AOR) at the 95% CI was used to identify the final associated factors. A significance level of 0.05 was taken as a cutoff value for all statistical significance tests. A two‐tailed test with *p* < 0.05 at the 95% CI was statistically significant. Multicollinearity was checked between each variable using the variance inflation factor. Accordingly, two variables were removed from multivariable logistic analyses (VIF = 9.95 & 11.5). A Pearson goodness‐of‐fit test was employed to evaluate the model's suitability^26^ because our data were categorical, and we found that all of the expected values were consistent with the data as observed. Area under the curve was also calculated from the ROC curve, and the result showed that all the variables have area under the curve value of greater than 0.5 of threshold to the left upper corner.

## 3 RESULTS

### 3.1 Sociodemographic characteristics of respondents

In the current study, a total of 414 (98%) respondents out of 422 respondents were participated. The reason of 2% nonresponse rate was because they gave incomplete data during the study. The mean age of the participants was 15 years with a standard deviation of ±2. The majority of respondents 71.5% (296/414) live with their parents. More than half of the respondents, 53.4% (221/414) were born in urban. Regarding the status of education of their parents, this study reported that the 25.8% (107) father and 112 (27.1%) of mother have attended primary education. By profession, the majority of respondents were born to 198 (47.8%) fathers and 150 (36.2%) mothers who were active farmers (Table 1).

**Table 1:** Sociodemographic characteristics of adolescent school girls in Ambo town, Ethiopia, 2021 (*n* = 414).

Sociodemographic characteristics	Frequency	%
*Age*		
14–15 years	199	48.1
16–18 years	215	51.9
*Student's educational status*		
Primary school	264	63.8
Secondary school	150	36.2
*Fathers' education status*		
Unable to read and write	73	17.6
Able to read and write	82	19.8
Primary education	107	25.9
Secondary education	62	15.0
College diploma and above	90	21.7
*Mothers' education status*		
Unable to read and write	101	24.3
Able to read and write	82	19.8
Primary education	112	27.1
Secondary education	76	18.4
College diploma and above	43	10.4
*Fathers' occupation*		
Merchant	42	10.1
Government employee	118	28.6
Private employee	38	9.2
Farmer	198	47.8
Others*	18	4.3
*Mothers' occupation*		
Merchant	58	14.0
Government employee	58	14.0
Private employee	44	10.6
Farmer	150	36.2
Housewife	102	24.6
Others*	2	0.5
*Place of birth*		
Urban	221	53.4
Rural	193	46.6

* Daily laborer, driver, religious leader.

### 3.2 Respondent's level of knowledge about CC, HPV infection, and HPV vaccine

More than three‐fourths of girls, 77.3% (320) had heard about CC and the HPV vaccine 78.7% (326), respectively. On the other hand, about 71.7% (297) know the method of prevention of CC. However, compared with this 57.2% (237) hadn't heard about HPV infection. Nearly three‐fourths 306 (73.9%) of respondents don't know recommended doses of HPV vaccine. The overall knowledge of HPV infection and HPV vaccine was 37/414 (8.9%) and 103/414 (24.9%), respectively. Moreover, the overall knowledge score about CC, HPV infection, and HPV vaccine was found to be very low 47/414 (11.4%) (Table 2).

**Table 2:** Cervical cancer, HPV infection, and HPV vaccination‐related knowledge of adolescent school girls in Ambo town, Ethiopia, 2021 (*n* = 414).

Variables		Category	Frequency	%
*Knowledge of cervical cancer*				
Have you ever heard cervical cancer		No	94	22.7
		Yes	320	77.3
Risk factors for cervical cancer		Don't know	386	93.2
		Know	28	6.8
Mode of transmission	Sexual transmission	No	248	59.9
		Yes	166	40.1
	Vertical transmission	No	398	96.1
		Yes	16	3.9
Signs and symptoms of cervical cancer		Don't know	325	78.5
		Know	89	21.5
Overall knowledge scores of cervical cancers		Poor	350	84.5
		Good	64	15.5
*Knowledge of HPV infection*				
Have you ever heard of HPV infection		No	237	57.2
		Yes	177	42.8
Who can contract HPV infection		Don't know	147	83.8
		Know	67	16.2
Diseases caused by HPV infection		Don't know	405	97.8
		Know	9	2.2
Risk factors for HPV infection		Don't know	353	85.3
		Know	61	14.7
Method of prevention of HPV infection		Don't know	392	94.7
		Know	22	5.3
Overall knowledge score toward HPV infection		Poor	377	91.1
		Good	37	8.9
*Knowledge of HPV vaccine*				
Have you ever heard about the HPV vaccine before		No	88	21.3
		Yes	326	78.7
The benefit of the HPV vaccine		Don't know	146	35.3
		Know	268	64.7
Who should get the HPV vaccination		Don't know	291	70.3
		Know	123	29.7
Recommended doses of HPV vaccine		Don't know	306	73.9
		Know	108	26.1
The ideal time HPV Vaccine is best recommended		Don't know	331	80.0
		Know	83	20.0
Overall knowledge score of the HPV vaccine		Poor	311	75.1
		Good	103	24.9
Overall knowledge score toward cervical cancer, HPV infection, and HPV vaccine		Poor	367	88.6
		Good	47	11.4

Abbreviation: HPV, human papillomavirus.

Many participants 331 (80%) responded that there was no community health education about the HPV virus. Even around 36.2% of participants reported that they did not get enough counseling about the benefits of the vaccination. About half 201/414 (48.6%) of those surveyed said that HPV vaccine promotion and health education are available in school. Most participants 283/414 (68.4%) reported that HPV vaccine promotions were available in their area.

### 3.3 Level of attitude of respondents toward HPV vaccination

Two‐thirds of the respondents, 312/414 (75.4%) have the view that the HPV vaccine helps to prevent HPV infection. The majority 301 (72.7%) of the respondents agreed that CC is a deadly disease. A large portion 302 (72.9%) of respondents agreed that HPV vaccination can minimize CC. Similarly, 302 out of 414 participants confirmed their own belief as the vaccine could save lives and improve health. Though, up to 286/414 (69.1%) stated their concern as having the HPV vaccine would possibly lead to sexual promiscuity, on the other hand, about 185 (44.7%) had a parental concern about having the vaccine. More than half 230 (55.6%) of the respondents have a favorable attitude toward HPV vaccinations (Table 3).

**Table 3:** Attitude toward HPV vaccination of adolescent school Girls in Ambo town, Ethiopia, 2021 (*n* = 414).

Variables	Attitude toward HPV vaccine			
	Unfavorable attitude		Favorable attitude	
	Frequency	%	Frequency	%
Cervical cancer is a deadly disease	113	27.3	301	72.7
The vaccination was beginning to minimize cervical cancer	112	27.1	302	72.9
Thinks vaccination helps to prevent HPV infection	102	24.6	312	75.4
Parental concern about having the vaccine	185	44.7	229	55.3
HPV vaccine saves lives and improves health	112	27.1	302	72.9
Whether you recommend the vaccine to others or not	75	18.1	339	81.9
Having the HPV Vaccine may become sexually promiscuous	128	30.9	286	69.1
Overall attitude	184	44.4	230	55.6

Abbreviation: HPV, human papillomavirus.

### 3.4 The perception of respondents toward CC, HPV infection, and HPV vaccination

The study revealed that 54% (224/414) of the respondents were found to have low perceived susceptibility; while 40% (166/414) has low perceived severity of the diseases. However, those who have a high perceived benefit of the HPV vaccine was about 66.5% (275/414). Especially, those who perceived getting vaccination can reduce the risk of CC was about 77% (319/414). Furthermore, possible side effects 66.9% (277/414), needle anxiety 61.6% (255/414), and a lack of knowledge of the HPV vaccine 57.2% (237/414) were the top reasons given for not wanting to have the vaccine (Table 4).

**Table 4:** Perception of the disease and the vaccine among adolescent school girls in Ambo town, Ethiopia, 2021 (*n* = 414).

	Poor perception		Good perception	
	Frequency	%	Frequency	%
*Perceived susceptibility*				
Any women are susceptible to facing cervical cancer	209	50.5	205	49.5
How much are you worried about contracting HPV infection	182	44	232	56
If you don't get vaccinated how likely is it that you'll become infected	280	67.6	134	32.4
*Perceived severity*				
Being infected with HPV would have major consequences on life	183	44.2	231	55.8
Being infected with HPV would be devastating	149	36	265	64
It would be very serious if someone became infected with HPV	167	40.3	247	59.7
*Perceived benefit*				
If you get vaccinated you can reduce your risk of cervical cancer	95	23	319	77
If you get vaccinated for HPV you can reduce the risk of vaginal cancer	113	27.3	301	72.7
Getting vaccinated will decrease your chances of getting genital warts	208	50.2	206	49.8
*Perceived barriers*				
Concerns about possible side effects	277	66.9	137	33.1
Needle phobia	255	61.6	159	38.4
Miss understanding about HPV vaccine	237	57.2	177	42.8
Lack of information	171	41.3	243	58.7
Overall perception	175	42.3	239	57.7

Abbreviation: HPV, human papillomavirus.

### 3.5 Factors associated with knowledge of respondents about HPV vaccination

Multiple logistic regression analysis showed that the educational status of the respondent and the existence of the source of information were associated with knowledge of HPV vaccination. Adolescents of secondary education status were two times more knowledgeable about HPV vaccination as compared with Adolescents of primary education level [AOR = 2.011, 95% CI; (1.239, 3.265)]. Respondents who have had Health workers as their main source of information had two times more likely to be knowledgeable about the HPV vaccine than those who didn't hear from health providers [AOR = 2.13, 95% CI; (1.304, 3.486)]. Additionally, respondents who have heard about the HPV vaccine at school were 1.6 times more likely to know about the HPV vaccine than those who didn't hear about it at school [AOR = 1.66, 95% CI; (1.02, 2.71)] (Table 5).

**Table 5:** Factors associated with knowledge of respondents toward HPV vaccination among adolescent school girls in Ambo town, Ethiopia, 2021 (*n* = 414).

Variables			Level of Knowledge		COR (95% CI)	AOR (95% CI)	*p* Value
			Good	Poor			
Educational level		Secondary school	48 (11.6)	102 (24.6)	1.79 (1.14, 2.82)	2.01 (1.24, 3.26)*	0.005
		Primary school	55 (13.3)	209 (50.5)	1.0	1.0	
Source of information	Health providers	Yes	59 (14.3)	111 (18.8)	2.42 (1.54,3.80)	2.13 (1.30, 3.48)*	0.003
		No	44 (10.6)	200 (48.3)	1.0	1.0	
	School	Yes	46 (11.1)	91 (21)	1.95 (1.23, 3.08)	1.66 (1.02, 2.71)*	0.04
		No	57 (13.8)	220 (53.1)	1.0	1.0	
	Internet	Yes	11 (2.7)	14 (3.4)	2.54 (1.12, 5.78)	2.21 (0.92, 5.31)	0.07
		No	92 (22.2)	297 (71.7)	1.0	1.0	

Abbreviations: AOR, adjusted odds ratio; COR, crude odds ratio; HPV, human papillomavirus.

* Statistically significant at *p* < 0.05 with COR and AOR.

### 3.6 Factors affecting respondents' attitudes about HPV vaccination

Adolescents who have heard about the HPV vaccine before vaccination had 3.4 times more favorable attitudes toward the vaccine than those who had never heard about it [AOR = 3.43, 95% CI; (1.34, 8.74)]. Moreover, those students who highly perceived the severity of the diseases were 2.9 times more likely to have a favorable attitude toward the vaccine than their counterparts [AOR = 2.89, 95% CI; (1.61, 5.21)]. Likewise, the Perceived benefit of the vaccine was 4.26 higher among Adolescents who have a favorable attitude toward the vaccine as compared with those who had unfavorable attitudes toward the vaccine [AOR = 4.26, 95% CI; (2.43, 7.48)]. On the other hand, respondents who strongly perceived the expected barriers were 50% less likely to have a favorable attitude as compared with those who were less likely to perceive the barriers [AOR = 0.5, 95% CI; (0.3, 0.84)]. Concerning the availability of promotion, adolescents who had available awareness creation promotion at their schools were 1.9 times more likely to have a positive attitude than those respondents who had no awareness creation promotion at their schools [AOR = 1.93, 95% CI; (1.06, 3.51)] (Table 6).

**Table 6:** Factors associated with attitude of respondents toward HPV vaccination among adolescent school girls in Ambo town, Ethiopia, 2021 (*n* = 414).

Variables		Attitude toward HPV vaccination		COR (95% CI)	AOR (95% CI)	*p* Value
		Favorable	Unfavorable			
Ever heard about HPV vaccine	Yes	273 (65.9)	53 (22.8)	8.15 (4.64, 14.4)	3.43 (1.341, 8.74)*	0.01
	No	32 (7.7)	56 (13.5)	1.0	1.0	
Perceived susceptibility	Good	167 (40.3)	32 (7.7)	2.16 (1.45, 3.22)	1.07 (0.6, 1.9)	0.83
	Poor	138 (33.3)	77 (18.6)	1.0	1.0	
Perceived severity	Good	215 (51.9)	31 (7.5)	4.19 (2.76, 6.36)	2.89 (1.61, 5.21)*	<0.001
	Poor	90 (21.7)	78 (18.8)	1.0	1.0	
Perceived barrier	Good	101 (24.4)	116 (28)	0.46 (0.31, 0.68)	0.5 (0.3, 0.84)*	0.009
	Poor	129 (31.2)	68 (16.4)	1.0	1.0	
Availability of awareness creation	Yes	173 (41.7)	28 (6.7)	3.28 (2.17,4.94)	1.93 (1.06, 3.51)*	0.03
	No	132 (31.8)	81 (19.5)	1.0	1.0	

Abbreviations: AOR, adjusted odds ratio; COR, crude odds ratio; HPV, human papillomavirus.

* Statistically significant at *p* < 0.05 with COR and AOR.

## 4 DISCUSSION

In this study, the proportion of Adolescent school girls who were knowledgeable about HPV vaccination was found to be only 24.9% (95% CI: 20.8–29.0). This was higher than two previous studies conducted on adolescent Girls in Latvia (9.9%) and Niger (11.4%).^3^, ^12^ However, the results from other studies in Puducherry (43.3%) and Uganda (47.7%) revealed better knowledge of HPV vaccination compared with the present study.^27^, ^28^ The variation observed can be explained by the differences in socioeconomic status, sample size, study settings, cultural norms, and access to information. This could be attributed to increased exposure to information regarding the HPV vaccine following the implementation of national HPV immunization, as well as various campaigns by both the public and private sectors.^29^

The current finding indicated that about 44.4% (95% CI: 39.6–49.5) of adolescents had unfavorable attitudes toward HPV vaccination, which is almost comparable with a study conducted in Lagos, Nigeria (44.2%).^30^ In contrast to our findings, another study from a developed country (Italy) showed that there were lower rates of unfavorable attitudes toward HPV vaccination (20%).^24^ The disparity could be explained by inequalities in socioeconomic class, educational level, and inadequate coverage of targeted educational programs (low access to information among low‐income countries.^29^, ^31^

Beyond assessing the prevalence rate, the study also pointed out factors that were associated with adolescent's knowledge and attitude toward the HPV vaccine. In this study, it was discovered that adolescent girls' knowledge about the HPV vaccine increased by about two times more than that of their counterparts when they had access to sources of information from health professionals and at school. This finding is consistent with another similar study from the United States, in which adolescents use school and health personnel as their primary sources of knowledge about HPV vaccine.^32^ This is because most schoolgirls perceive health workers and school staff to be more knowledgeable about the vaccine than any other career, which is why the information provided by schools and health experts is reliable to them.

This study also found that one of the factors associated with the knowledge of HPV vaccination was an adolescent's level of education. Those girls from secondary school were more knowledgeable than those from primary school. The finding was consistent with a finding from a similar study conducted on Mozambican and Nepali adolescents.^33^, ^34^ The reason might be because the students in high school were more likely to learn and read about HPV and CC. As a result, the CC education that was included in the Grade 10 curriculum supports the students to have an awareness of HPV vaccination.^35^

Also, the results of this study showed that adolescents who had heard about the HPV vaccine (who had been exposed to information) before immunization had a more favorable attitude toward it. The result of this study is in line with the study conducted in Italy.^24^ This is explained by the fact that, having preinformation about the vaccine helps in recognizing the benefits of the vaccine which in turn helps to develop a positive outlook for a vaccine.

Another finding of this study indicated that adolescents who perceived the severity of the diseases well have had a more positive attitude toward the vaccine than who did not perceived the severity of the HPV virus. The result of this study is also in line with a piece of evidence from the study conducted on adolescent daughters in Hong Kong and Kenya, in which perceived severity was strongly associated with a positive attitude toward the HPV vaccine.^12^, ^36^ In addition to this, the perceived benefit of the vaccine could make to have a more positive attitude toward the vaccine than those who do not believe in the benefit of the vaccine. Not only this but also, the result of this study was similar to a study conducted in Kenya in which perceived health benefits were strongly associated with a positive attitude toward the HPV vaccine.^36^ This may be because understanding the vaccine's advantages may contribute to developing a favorable opinion of immunization.

Another individual‐level factor was perceived barrier, in which those who strongly perceived different barriers as it retains them from vaccination were less likely to have positive belief toward the HPV vaccine. This can be explained by the fact that individual barriers to vaccine acceptance such as fear of needle injection, side effects as well as broader sociocultural context can negatively affect attitude toward the HPV vaccination.^37^ Another possible reason might be due to little existence of promotion and availability of information toward HPV vaccine.

The current study didn't address all components of perception which might be literarily well addressed by the health belief model. This study did not use coefficient plot diagram to illicit attitude and perception. There might be also social desirability bias. Since the study was limited to school facilities, it was difficult to generalize to all Adolescents of similar age groups living in the same district. Thus, to minimize the effect of the aforementioned limitations scholars with similar interests are recommended to conduct a community‐based study and use this study considering the identified limitations of the study.

## 5 CONCLUSION AND RECOMMENDATION

The success of the newly recommended HPV vaccination will be determined by adolescents knowledge and attitude. According to the findings of this survey, the majority of respondents have poor knowledge and an unfavorable attitude toward the HPV vaccine. This implies that lack of awareness about the HPV vaccine, as well as negative attitudes toward it, are barriers to immunization and may lead to uncertainty or resistance to vaccination. Therefore, increasing the awareness and improving the attitudes of adolescent school girls on HPV vaccination through school clubs, Mini‐media, and health educations at schools and health facilities should be strengthened. Moreover, efforts must be made to enhance adolescent girls' perceptions of the HPV vaccine by educating them about CC, and HPV infection.

## AUTHOR CONTRIBUTIONS

**Ephrem Yohannes**: Conceptualization; data curation; formal analysis; funding acquisition; methodology; software; supervision; validation; writing—original draft; writing—review and editing. **Mulugeta W. Beyen**: Conceptualization; investigation; methodology; supervision. **Gizachew A. Bulto**: Formal analysis; methodology; validation; writing—original draft. **Eshetu E. Chaka**: Formal analysis; project administration; software; validation. **Bikila T. Debelo**: Software; visualization; writing—original draft. **Maru M. Erena**: Data curation; formal analysis; funding acquisition. **Tefera L. Tasu**: Conceptualization; formal analysis; project administration; validation; writing—review and editing.

## CONFLICT OF INTEREST STATEMENT

The authors declare no conflict of interest.

## ETHICS STATEMENT

The ethical approval was obtained from the Institutional Review Board (IRB) of the College of Medicine and Health Sciences, Ambo University with the Reference number AU/PGC/29/2020. Verbal and written informed consent were obtained separately from parents to ensure their legal preparation on behalf of their children for adolescents aged 18 and below. To maintain the confidentiality of information provided by study subjects, the data collection process is anonymous. Participation is voluntary and withdrawal from the study at any time was considered. No name or other identifying information was included in the questionnaire. We are confirming all the information involving the participants was following the guidelines of an institutional review board of Ambo University, college of medicine and health sciences.

## TRANSPARENCY STATEMENT

The lead author Ephrem Yohannes affirms that this manuscript is an honest, accurate, and transparent account of the study being reported; that no important aspects of the study have been omitted; and that any discrepancies from the study as planned (and, if relevant, registered) have been explained.

## Data Availability

The data sets used and/or analyzed during the current study are available from the corresponding author upon written request.
